# Lobectomy for Lung Cancer in a Patient With a History of Acute Exacerbation of Interstitial Lung Disease

**DOI:** 10.1016/j.atssr.2025.08.013

**Published:** 2025-09-05

**Authors:** Satoshi Koezuka, Yoko Azuma, Megumi Kusano, Shumpei Kato, Kaiki Tamori, Susumu Sakamoto, Naobumi Tochigi, Akira Iyoda

**Affiliations:** 1 Division of Chest Surgery, Department of Surgery, Toho University School of Medicine, Tokyo, Japan; 2 Department of Respiratory Medicine, Toho University School of Medicine, Tokyo, Japan; 3 Department of Surgical Pathology, Toho University School of Medicine, Tokyo, Japan

## Abstract

Acute exacerbation (AE) of interstitial lung disease (ILD) occurring after lung cancer surgery is a life-threatening complication. A history of AE before the surgery is strongly associated with postoperative AE. We report surgery for lung cancer in a patient with a history of AE of ILD associated with microscopic polyangiitis. We successfully performed a right middle lobectomy for lung cancer 7 months after AE of ILD. We revealed that surgical techniques and perioperative management for preventing AE might prevent postoperative complications.

Microscopic polyangiitis (MPA) is a systemic vasculitis characterized by the presence of antineutrophil cytoplasmic antibody (ANCA). MPA is frequently associated with interstitial lung disease (ILD),^1^ and MPA has been linked to the risk of lung cancer.^2^ Treatment of lung cancer concomitant with ILD carries the risk of acute exacerbation (AE) of ILD.^3^ Here, we report a patient with a history of AE of MPA associated ILD (MPA-ILD) who underwent surgery for lung cancer.

A 59-year-old man with MPA-ILD who was undergoing treatment with prednisolone (15 mg daily) was referred to the Department of Respiratory Medicine in our hospital for evaluation of a lung tumor. The patient had a history of nonspecific interstitial pneumonia diagnosed by way of a thoracoscopic lung biopsy specimen 10 years previously, and an AE 7 months previously treated by cyclophosphamide pulse therapy and 50 mg prednisolone. After stabilization of interstitial pneumonia, prednisolone was tapered to 15 mg. No other treatment was administered for ILD.

Blood testing showed an elevated levels of sialyl-Lewis X (SLX; 43.9 ng/mL [normal <5.0 ng/mL]), Krebs von den Lungen-6 (KL-6; 2216 U/mL [normal <500 U/mL]), and MPO-ANCA (15.2 U/mL [normal <3.5 U/mL]). Chest computed tomography showed bilateral reticular opacities in the subpleural basal segments of the pulmonary lobes (Figure 1A) and a 71-mm partially solid tumor (solid component, 36 mm) in the right middle lobe (Figure 1B). A transbronchial biopsy specimen was diagnosed as primary adenocarcinoma (clinical T2a N0 M0 stage IB).

**Figure 1:**
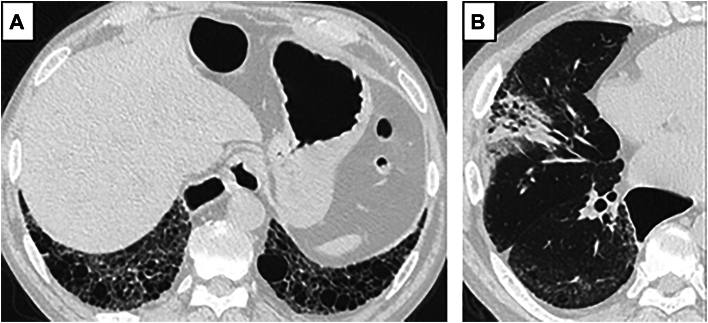
Computed tomography shows (A) a nonspecific interstitial pneumonia pattern and (B) a partially solid tumor in the right middle lobe.

The patient was referred to surgery. Pulmonary function testing showed the following values for vital capacity percentage, forced expiratory volume in 1 second, forced expiratory volume in 1 second percentage, and percentage of diffusion capacity of the lung for carbon monoxide : 91.0%, 2.49 L, 70.1%, and 50.8%, respectively. Room-air blood gas values were Pao_2_ 86.4 torr and Paco_2_ 34.0 torr. The 6-min walk test was 410 m, and the minimum oxygen saturation by pulse oximetry was 84%.

The risk scores for postoperative AE in a patient with ILD^4^ were history of AE, 5 points; surgical procedures, 4 points; male sex, 3 points; preoperative steroid use, 3 points; and KL-6, >1000 mL/U, 2 points (total risk score, 17 points). The predicted incidence of postoperative AE was 44.5% (high-risk group); however, the patient desired surgery. One month presurgery he received respiratory physiotherapy. Perioperatively, prednisolone 15 mg was continued, and no drugs were administered to prevent postoperative AE.

A right middle lobectomy with hilar lymph node dissection was performed by video-assisted thoracoscopy. A camera port was inserted at the eighth intercostal space, and an open chest wound extending 8 cm laterally was created in the fifth intercostal space. The lung collapsing was poor, and especially the lower lobe tissue appeared fragile, with pleural coarsening and sclerotic changes (Figure 2A).

**Figure 2:**
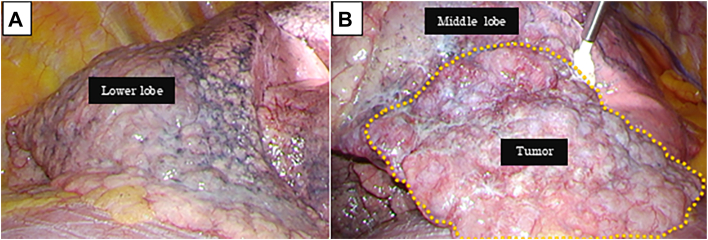
(A) The visceral pleura of the lower lobe showed surface roughness and sclerotic changes. (B) The tumor was located in the middle lobe (yellow dotted circle).

The tumor was located in the middle lobe and appeared to invade the pleura (Figure 2B). Interlobular fissure formation was performed using ECHELON ENDOPATH Staple Line Reinforcement (Ethicon Endo-Surgery, Johnson & Johnson, Ltd). The regenerative oxidized cellulose membrane and fibrin glue were used for reinforcement of staple lines and the bronchial stump. Operative time was 175 min, and volume of blood loss was 177 mL. During surgery, we took care of protection for preserving upper and lower lobes, operation time, sealing for lymphatic vessels, or stopping air leakage of the lung. The anesthesiologist administered low-concentration oxygen and low-pressure ventilation.

The histopathologic diagnosis of the tumor was invasive mucinous adenocarcinoma (pathological T4 N0 M0 stage IIIA) (Figure 3).

**Figure 3:**
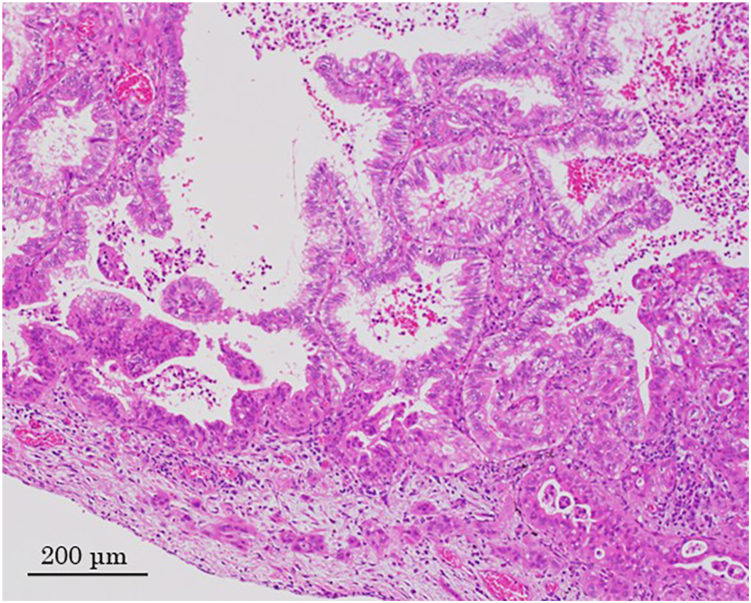
Microscopic examination shows invasive mucinous adenocarcinoma (hematoxylin and eosin staining, original magnification ×10).

The thoracic drain was removed on postoperative day 1, and the patient underwent breathing exercises and walking exercises to prevent pneumonia. He was discharged on postoperative day 7, with no evidence of an AE of ILD. At 9 months after surgery, the tumor had metastasized to the segmental station #13 in the right lower lobe, right pleura, bilateral lower pulmonary lobes, and bone. The patient underwent 3 courses of nanoparticle albumin–bound-paclitaxel plus carboplatin chemotherapy and died 15 months after surgery.

## Comment

AE of ILD that develops after lung cancer surgery is a fatal complication. A multi-institutional cohort study reported a 43.9% mortality rate for AEs occurring after surgery.^5^ A history of AE before the surgery was identified as the strongest independent risk factor. A study of surgical outcomes for lung cancer patients with concomitant ILD with a history of AE found that postoperative AEs developed in 35.3% of patients.^4^ Here we report surgery for a lung cancer in a patient with MPA-ILD with a history of AE.

The annual incidence of AEs in patients with MPA-ILD is 7.2%,^6^ which is comparable to the 5% to 15% annual incidence of AEs occurring in patients with idiopathic pulmonary fibrosis. The occurrence of AE in MPA-ILD patients can be fatal; the reported median survival after an AE is 22 days.^6^ ANCA-mediated neutrophil overactivation induced by infection or nonspecific inflammation is an important factor involved in the exacerbation of ANCA-associated vasculitis. ANCA is directly involved in the development and progression of MPA. ANCA is directly associated with pulmonary fibrosis, because of associated oxidation products that trigger the proliferation of fibroblasts and release of proteolytic enzymes, with subsequent injury to pulmonary tissue.^1^^,^^7^

The prevention of postoperative AE necessitated a surgical approach and perioperative management that minimized the inflammatory response. A miniopen thoracoscopic approach has sometimes been used to perform short and minimally invasive surgical procedures in patients with ILD whose lungs were difficult to collapse. Unnecessary contact with the lung and strong pressure applied to lung tissue should be avoided to minimize the production of inflammatory cytokines.

Intraoperatively, we collaborated with the anesthesiologist to avoid high concentrations of oxygen, which can damage lung tissue by stimulating the production of active enzymes and inflammatory cytokines. Iyoda and colleagues^8^ reported that postoperative episodes of inflammation were triggers for AE and concluded that special attention should be paid to postoperative pneumonia prevention. To prevent pneumonia in our patient, respiratory physiotherapy was performed preoperatively, and sampling of the mediastinal lymph nodes was performed so the thoracic drain could be removed early to start respiratory physiotherapy as soon as possible. We planned to perform selective lymph node dissection if our sampled lymph nodes were positive for malignancy. Even with various modifications, the development of AE cannot be completely prevented, so careful postoperative follow-up is necessary to detect early signs of AE so that treatment can be initiated immediately.

In conclusion, we successfully performed lobectomy for lung cancer in a high-risk patient with a predicted incidence of postoperative AE of 44.5%. Surgical techniques and perioperative management for the suppression of inflammation may aid the prevention of postoperative AE.
